# Effect of successful revascularization on left ventricular diastolic dysfunction in patients with aortoiliac occlusive disease

**DOI:** 10.1097/MD.0000000000012339

**Published:** 2018-09-21

**Authors:** Wonho Kim, Tae Soo Kang

**Affiliations:** a Division of Cardiology, Eulji University Hospital, Eulji University School of Medicine, Daejeon; b Division of Cardiology, Dankook University Hospital, Dankook University College of Medicine, Cheon-an, Chungchungnam-do, Republic of Korea.

**Keywords:** aortoiliac occlusive disease, diastolic dysfunction, peripheral artery intervention

## Abstract

Aortoiliac occlusive disease (AIOD) affects the systemic vascular resistance and increases the afterload because the left ventricle (LV) must work harder to eject blood into a smaller vascular bed. This study was to determine whether successful revascularization of AIOD is associated with improvement of left ventricular diastolic dysfunction (LVDD).

A total of 37 patients with AIOD (34 men and 3 women; age 65.1 ± 7.2 years) were analyzed. The primary endpoint was defined as the change in the mitral *E*/*E*′ ratio.

There were no significant changes in the *E* velocity (from 0.7 ± 0.2 to 0.7 ± 0.2 m/s, *P*-value = .153), *A* velocity (from 0.8 ± 0.2 to 0.9 ± 0.2 m/s, *P*-value = .169), LAVI (from 36.1 ± 18.7 to 33.9 ± 15.7 mL/m^2^, *P*-value = .176), *E*/*A* ratio (from 0.9 ± 0.4 to 0.8 ± 0.2, *P*-value = .091), and *E*′ velocity (from 6.5 ± 2.0 to 6.9 ± 2.1 m/s, *P*-value = .068). However, successful revascularization significantly reduced the *E*/*E*′ ratio (from 14.1 ± 5.7 to 11.7 ± 3.3, *P*-value = .015). Additionally, a significant increase in the *A*′ velocity (from 9.1 ± 1.9 to 10.0 ± 2.2 m/s, *P*-value = .029) and a decrease in the LA diameter (from 40.7 ± 6.4 to 38.6 ± 6.6 mm, *P*-value = .014) were noted.

Our results show that a successful revascularization of AIOD was associated with an improved *E*/*E*′ ratio.

## 1 Introduction

Left ventricular diastolic dysfunction (LVDD) is usually the result of impaired LV (left ventricular) relaxation with or without reduced restoring forces and increased LV chamber stiffness, which increases the cardiac filling pressure.^[1]^ When treating a patient with LVDD, it is essential to reduce the cardiac preload and afterload to optimize the hemodynamics.^[2]^ On the other hand, a recent study highlighted a high prevalence of LVDD in patients with peripheral artery disease (PAD).^[3]^ In particular, aortoiliac occlusive disease (AIOD) may result in the progression of LVDD through LV remodeling secondary to an increased afterload.^[4]^ Considering that systemic vascular resistance (SVR) is often used as a measure of the afterload in a clinical setting, severe AIOD might affect the SVR and subsequently increase the afterload because the LV must work harder to eject the blood into the occluded iliac artery.^[5]^ It remains unknown in the published literature whether successful revascularization of severe AIOD improves LVDD, and if so, whether its effect is attributable to the attenuation of LVDD. The current study investigated the effect of successful revascularization on LVDD in patients with AIOD.

## 2 Methods

### 2.1 Study population, identification of patients

The study population was retrospectively recruited from consecutive patients who were admitted at 2 university hospitals (between January 2005 and September 2017) and discharged after successful percutaneous revascularization of severe AIOD. The list of these patients was matched with a list of patients with pre-procedural and post-procedural echocardiograms around the time of the revascularization procedure. Patients were excluded if they had a history of acute myocardial infarction or admission due to heart failure (HF) aggravation before checking the follow-up echocardiogram after the revascularization procedure, evidence of critical limb ischemia (CLI) with poor tibial runoff vessels, significant LV hypertrophy at baseline (LV wall thickness ≥13 mm), significant valve diseases (more than or equal to moderately severe valve diseases), and atrial arrhythmia including atrial fibrillation.

This study was conducted in accordance with the Declaration of Helsinki and was approved by our institutional review board.

### 2.2 Study end points

The primary endpoint was defined as the change in the mitral *E*/*E*′ ratio, an index of LVDD. The secondary endpoints included the changes in the pulsed-wave Doppler (PWD)-derived trans-mitral filling indices (*E*- and *A*- velocities, *E*/*A* ratio, and deceleration time [DT]), Tissue Doppler imaging (TDI) derived indices (*E*′-, *A*′-, and *S*′ velocities, *E*/*E*′ ratio), left ventricular ejection fraction (LVEF), left atrial volume index (LAVI), and improvement on LVDD.

### 2.3 Echocardiographic evaluation and definitions

The left atrial (LA) diameter was measured in the parasternal long axis view, and the end-systolic LAVI was measured with the biplane area-length method.^[6]^ The PWD-derived trans-mitral velocities were obtained at the mitral leaflet tips.^[7]^ TDI derived indices were acquired in the apical 4-chamber view with the sample positioned at the septal and lateral mitral annulus for determination of the systolic (*S*′), early diastolic (*E*′), and late diastolic (*A*′) velocities. For all parameters, the average of 3 consecutive heartbeats was recorded, and the velocities were recorded at end-expiration. The *E*′ velocity was determined at each annular site and averaged. The PWD-derived *E* velocity /TDI-derived *E*′ velocity (*E*/*E* ratio) was calculated to determine the LV filling pressure. The systolic function was evaluated by LVEF using the modified Simpson rule from the biplane 4-chamber and long-axis views. LVDD refers to a disturbance in ventricular relaxation, distensibility or filling regardless of whether the LVEF is normal or depressed and whether the patient is asymptomatic or symptomatic.^[8]^ According to the recently published guideline, LVDD was considered to be present if more than half of the 4 recommended parameters meet the cutoff values for identifying abnormal function.^[1]^ Briefly, the 4 recommended parameters and their abnormal cutoff values are the annular *E*′ velocity (septal *E*′ < 7 cm/s, lateral *E*′ < 10 cm/s), average *E*/*E*′ ratio >14, maximum LAVI >34 mL/m^2^, and peak TR velocity >2.8 m/s. Improvement of LVDD was defined as any decrease of the baseline *E*/*E*′ ratio or any increase of the baseline *E*′ velocity after the revascularization procedure.^[9]^

Severe AIOD was defined as a total or subtotal occlusion of the aortoiliac segment (TASC-II C or D lesions) with several visceral and parietal collateral channels.

Coexistent coronary artery disease (CAD) was defined as the presence of any degree of narrowing in at least 1 major coronary arteries on angiogram or computed tomography in the study population.

Successful revascularization was defined as revascularization of the occlusive lesion with <30% residual diameter stenosis and absence of dissection impairing blood flow at the final angiographic control.

### 2.4 Statistical analysis

The mean (standard deviation) and percentages are reported for continuous and categorical variables, respectively. Logistic regression analysis was performed to identify possible predictors of improvement of LVDD. A *P*-value of <.05 was considered to be statistically significant. The analyses were performed using the SPSS version 13 software (SPSS, Inc.).

## 3 Results

**Figure 1:**
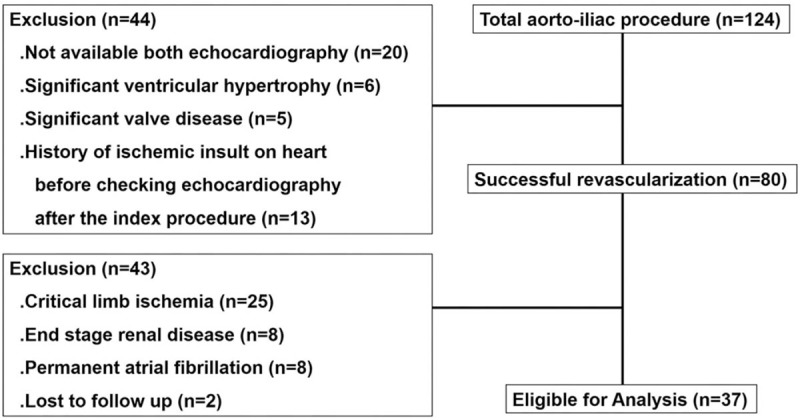
Flow chart showing patient selection.

We identified 80 patients who underwent an endovascular intervention for AIOD. With careful review of the exclusion criteria, 37 patients (34 men and 3 women; age 65.1 ± 7.2 years) were included in the analysis (Fig. 1). The characteristics of the study population are shown in Table 1. Thirty one patients (83.8%) had hypertension; 23 patients (62.2%) had diabetes, and 23 (62.2%) had a history of smoking. Twenty-one patients (56.8%) had unilateral iliac artery disease, whereas 16 patients (43.2%) had bilateral iliac artery disease. Baseline echocardiogram was performed 23.7 ± 36.7 days before the revascularization procedure, and the interval between the revascularization procedure and follow-up echocardiograms averaged 93.2 ± 66.7 days. Medications at the time of admission included diuretics in 9 patients (24.3%), beta-blockers in 20 patients (54.1%), a renin-angiotensin-aldosterone system (RAAS) inhibitor in 16 patients (43.2%), and a calcium channel blocker (CCB) in 11 patients (29.7%).

**Table 1:**
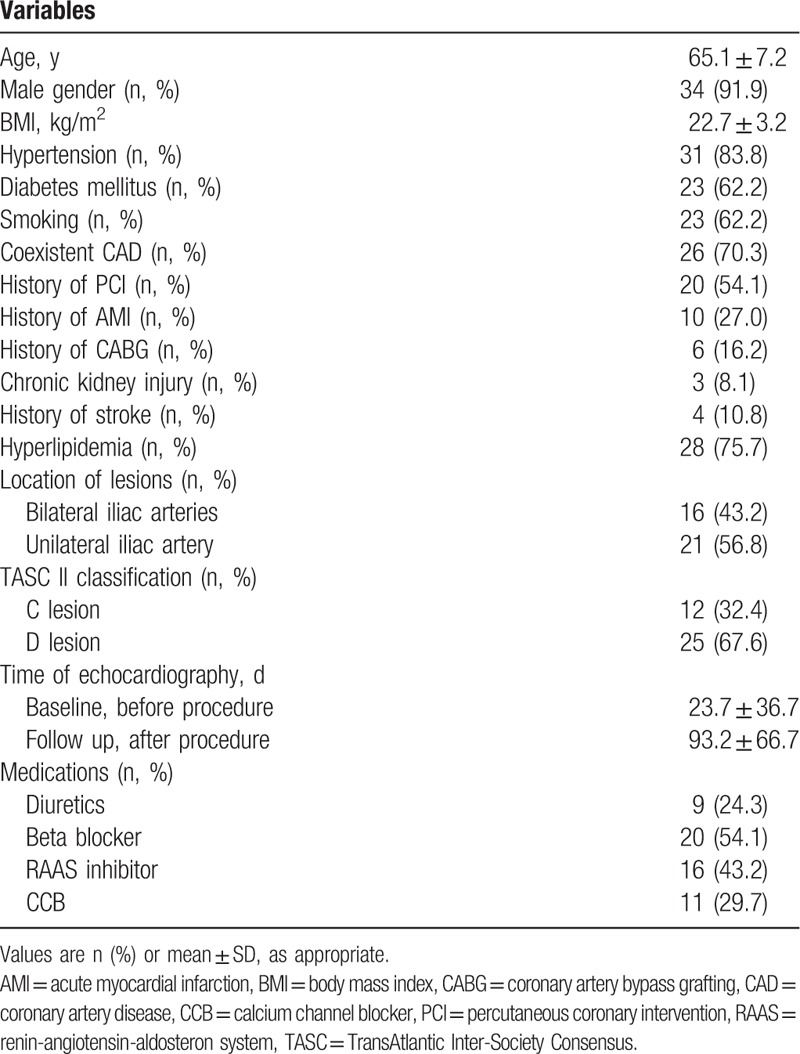
Patient baseline characteristics.

**Figure 2:**
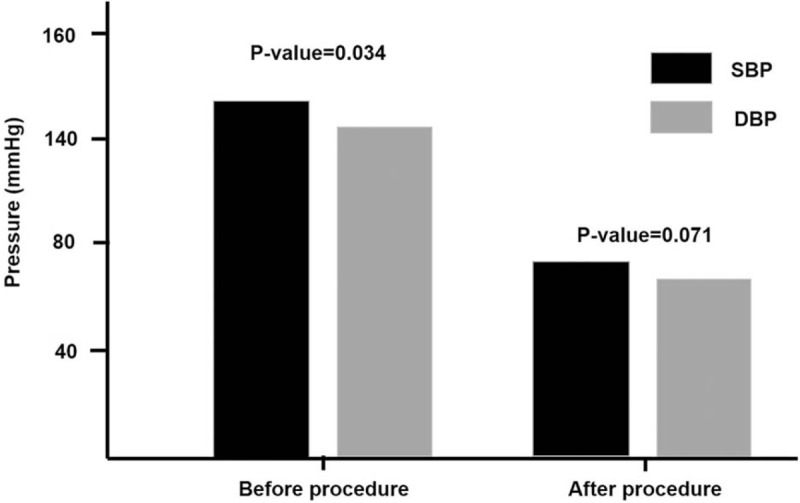
The change of SBP and DBP by successful revascularization on AIOD.AIOD = aortoiliac occlusive disease, DBP = diastolic blood pressure, SBP = systolic blood pressure.

The effect of revascularization procedure on reducing systolic blood pressure was significant (from 135.43 ± 22.54 to 125.70 ± 17.98 mmHg, *P*-value = .034). However, the change of diastolic blood pressure was not different (from 73.86 ± 10.28 to 69.59 ± 9.92 mmHg, *P*-value = .071) (Fig. 2).

**Figure 3:**
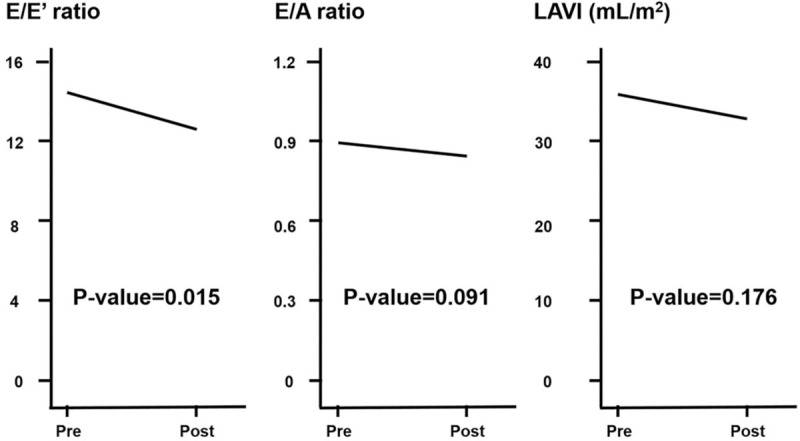
Effect of successful revascularization on diastolic function estimated by echocardiography. Echocardiography was performed 23.7 ± 36.7 days before the index procedure, and the interval between the index procedure and follow-up averaged 93.2 ± 66.7 days. Graphs show the quatitative analyses of *E*/*E*′, *E*/*A*, and LAVI. LAVI = left atrial volume index.

Changes in the echocardiographic parameters as a result of successful revascularization are shown in Table 2. There were no significant changes in the *E* velocity (from 0.7 ± 0.2 to 0.7 ± 0.2 m/s, *P*-value = .153), *A* velocity (from 0.8 ± 0.2 to 0.9 ± 0.2 m/s, *P*-value = .169), DT (from 208.3 ± 47.5 to 217.8 ± 43.1 m/s, *P*-value = .537), and LAVI (from 36.1 ± 18.7 to 33.9 ± 15.7 mL/m^2^, *P*-value = .176). The *E*/*A* ratio, the most commonly used diastolic function parameter in the clinical setting did not yield a significant change (from 0.9 ± 0.4 to 0.8 ± 0.2, *P*-value = .091). The *E*′ velocity showed no significant changes during the study period (from 6.5 ± 2.0 to 6.9 ± 2.1 m/s, *P*-value = .068). However, successful revascularization significantly reduced the *E*/*E*′ ratio (from 14.1 ± 5.7 to 11.7 ± 3.3, *P*-value = .015) (Fig. 3). In addition, a significant increase in the *A*′ velocity (from 9.1 ± 1.9 to 10.0 ± 2.2 m/s, *P*-value = .029) and a decrease of the LA diameter (from 40.7 ± 6.4 to 38.6 ± 6.6 mm, *P*-value = .014) were noted. The highest decrease of the *E*/*E*′ ratio was observed in a 67-year-old woman with totally occluded bilateral iliac arteries (from 35.1 at baseline to 15.3 at follow up).

**Table 2:**
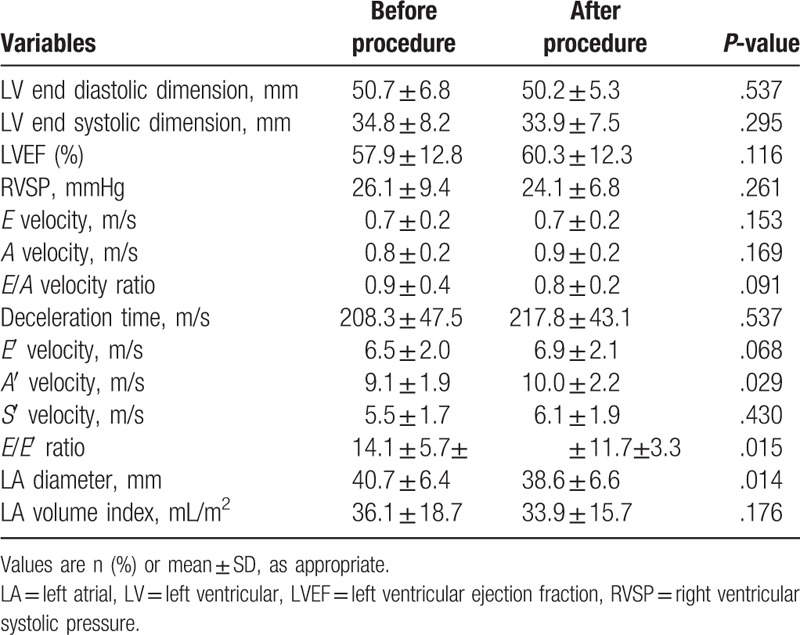
Two-dimensional echocardiographic and Doppler variables (paired *t* test).

**Figure 4:**
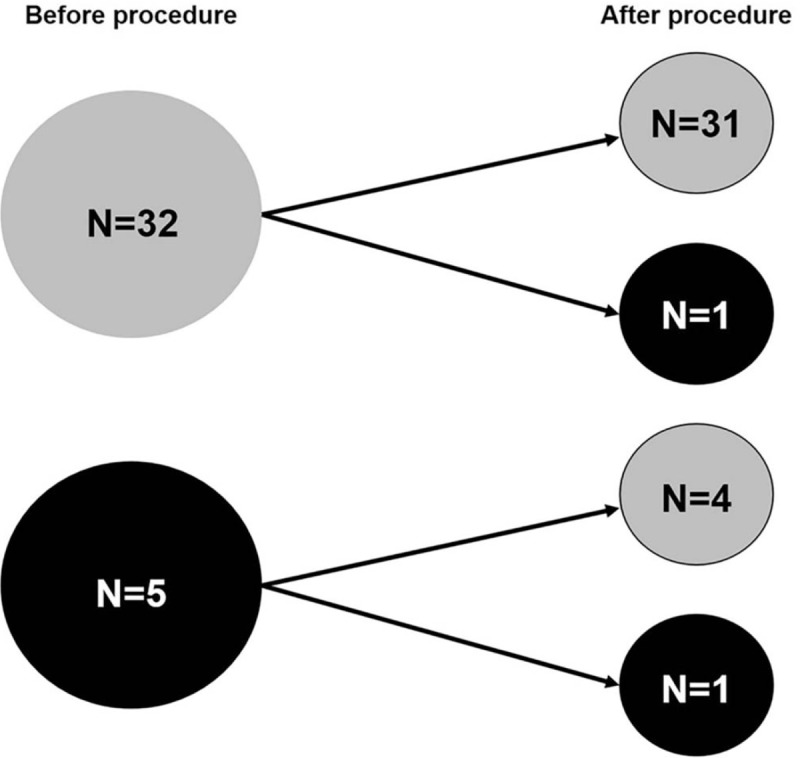
Baseline and follow up diastolic dysfunction. Gray indicates “relaxation abnormality”; Black, “pseudonormalization.”

When it comes to the grade of LVDD, baseline LVDD was present in all patients, with “Grade II, relaxation abnormality” being the most prevalent (86.5%, n = 32) and “Grade III, pseudonormalization” in only 13.5% (n = 5). On follow up echocardiogram, 2 patients (5.4%) had worsening, and 31 patients (83.8%) had no change, and 4 patients (10.8%) had improved baseline LVDD (Fig. 4).

Improvement of LVDD was observed in 25 patients (67.6%). Logistic regression analysis was performed to identify possible predictors of improvement of LVDD. However, we did not observe any significant parameters related to the improvement of LVDD; thus, multivariate analysis was not done.

## 4 Discussion

In the present study, we showed that the *E*/*E*′ ratio substantially decreased after successful revascularization in patients with AIOD. Changes in other parameters such as the *E* velocity, *E*′ velocity, *E*/*A* ratio, LVEF, and LAVI were not significant.

Echocardiogram has been used as the most important clinical tool in terms of evaluating LVDD. Even though the PWV-derived indices, in particular the *E*/*A* ratio, have been the most widely used to evaluate LVDD in the clinical setting, they however are largely influenced by cardiac preload.^[10]^ In contrast, TDI derived indices are known to be a more sensitive tool than the PWV-derived indices for the detection of afterload-related LVDD. The *E*/*E*′ ratio is the most reproducible TDI-derived parameter to estimate the LV filling pressure related to LVDD and is the preferred prognostic parameter in patients with LVDD. Although the change in the *E*′ velocity was not significant, it certainly was the improvement of the *E*′ velocity (from 6.5 ± 2.0 to 6.9 ± 2.1 m/s, *P*-value = .068) that contributed to the decreased *E*/*E*′ ratio after the successful revascularization procedure because the *E* velocity did not change at all (from 0.7 ± 0.2 to 0.7 ± 0.2 m/s, *P*-value = .153). Afterload reduction followed by a decrease of the LV filling pressure in conjunction with a successful revascularization procedure seems to be the main facilitating mechanism for our result.

AIOD is often associated with overt atherosclerosis involving CAD, which may cause subsequent heart failure. Theoretically, elevated aortic stiffness due to an occlusion of the iliac artery increases the LV afterload, and a high pulse pressure impairs the coronary blood flow resulting in hypertension, LV hypertrophy, LVDD, and ultimately overt HF.^[11,12]^ Overall, LVDD is more frequent in patients with AIOD, and the outcome data for this group are very limited. It is most likely, however, that this combination is associated with increased CV morbidity and mortality. Afterload is defined as the force that opposes shortening of the myocardial fibers. The most common descriptor of afterload in the clinical setting is the SVR. Thus, successful revascularization of AIOD may be an attractive method to improve LVDD by its afterload reducing effect. Given these results, it appears that successful revascularization of AIOD may have a beneficial effect on cardiac diastolic function leading to an improved prognosis independent of the symptomatic claudication relieving effect.

A recent study demonstrated the beneficial effect of revascularization on improving LVDD in patients with femoropopliteal (FP) artery disease.^[9]^ The circulation distal to an occluded lesion often undergoes collateralization, which reduces the SVR and thereby maintains a normal resting blood flow despite a reduced perfusion pressure.^[13]^ In particular, intact deep femoral artery (DFA) in patients with FP artery disease usually have good collateralization with the smallest increases in the afterload following FP artery occlusion. Thus, the greatest benefit on LVDD from the revascularization procedure would be anticipated in patients with AIOD rather than patients with FP artery disease.

One might wonder if medical therapy affects LVDD and works as a confounding factor, since the pharmacological actions of a RAAS inhibitor, diuretic or beta-blocker may affect LVDD. Decreases in the heart rate with a beta-blocker may affect the LVDD properties by increasing the LV relaxation rate.^[14]^ A RAAS inhibitor and diuretic may change the LV end diastolic volume and end systolic volume, resulting in an altered LV relaxation rate.^[15]^ However, these medications in this study had been started the day of the procedure or before, and little changed during the study period.

In the present study, the effect of revascularization procedure on reducing systolic blood pressure was significant. A large artery occlusion produces a strong reflection site which can develop abnormal or premature wave reflection and increase arterial stiffness. This plays an important role in the genesis of hypertension.^[15]^ This finding further underscores the potential important value of successful revascularization of AIOD in terms of blood pressure reduction.

There are several limitations to the present study. First, the number of study patients might have been small to detect the differences. The sample size may therefore be underpowered and accordingly the clinical implications may be limited. Second, we only obtained baseline and follow-up diastolic parameters and therefore, cannot clarify the variability of the diastolic parameters during the course of the study. There may be concern that the diastolic parameters could become altered according to the time of the echocardiographic assessment, which was not standardized in this study. Third, we did not take the length and diameter of the occluded lesion into consideration leading to a heterogeneous sample. Fourth, the pharmacological actions of a RAAS inhibitor, diuretic or beta-blocker may affect LVDD. Decreases in the heart rate with a beta-blocker may affect the LVDD properties by increasing the LV relaxation rate.^[14]^ A RAAS inhibitor and diuretic may change the LV end diastolic volume and end systolic volume, resulting in an altered LV relaxation rate. Meanwhile, of note is that patients with PAD including AIOD but no clinical evidence of CAD have the same relative risk of death from cardiac causes as those whose main diagnosis is CAD.^[16]^ Current recommendations for the treatment of LVDD primarily include control of underlying comorbidities such as hypertension, ventricular rate in atrial fibrillation, pulmonary congestion, and peripheral edema.^[17,18]^ Based on the result of this study, we suggest that successful revascularization of severe AIOD might be a possible way to reduce the afterload and to improve LVDD.

## 5 Conclusions

This study shows that successful revascularization of AIOD was associated with the improvement of the *E*/*E*′ ratio, and these changes may represent its effect on the afterload reduction.

## Author contributions

**Conceptualization:** Wonho Kim, Tae Soo Kang.

**Data curation:** Wonho Kim, Tae Soo Kang.

**Formal analysis:** Wonho Kim, Tae Soo Kang.

**Funding acquisition:** Wonho Kim.

**Investigation:** Wonho Kim, Tae Soo Kang.

**Methodology:** Wonho Kim.

**Project administration:** Wonho Kim.

**Resources:** Wonho Kim, Tae Soo Kang.

**Supervision:** Tae Soo Kang.

**Writing – original draft:** Wonho Kim, Tae Soo Kang.
